# Antimicrobial activity, improved cell selectivity and mode of action of short PMAP-36-derived peptides against bacteria and *Candida*

**DOI:** 10.1038/srep27258

**Published:** 2016-06-02

**Authors:** Yinfeng Lyu, Yang Yang, Xiting Lyu, Na Dong, Anshan Shan

**Affiliations:** 1 Laboratory of Molecular Nutrition and Immunity, Institute of Animal Nutrition, Northeast Agricultural University, Harbin, Heilongjiang, P.R. China

## Abstract

Antimicrobial peptides (AMPs) have recently attracted a great deal of attention as promising antibiotic candidates, but some obstacles such as toxicity and high synthesis cost must be addressed before developing them further. For developing short peptides with improved cell selectivity, we designed a series of modified PMAP-36 analogues. Antimicrobial assays showed that decreasing chain length in a certain range retained the high antimicrobial activity of the parental peptide and reduced hemolysis. The 18-mer peptide RI18 exhibited excellent antimicrobial activity against both bacteria and fungi, and its hemolytic activity was observably lower than PMAP-36 and melittin. The selectivity indexes of RI18 against bacteria and fungi were improved approximately 19-fold and 108-fold, respectively, compared to PMAP-36. In addition, serum did not affect the antibacterial activity of RI18 against *E. coli* but inhibited the antifungal efficiency against *C. albicans*. Flow cytometry and electron microscopy observation revealed that RI18 killed microbial cells primarily by damaging membrane integrity, leading to whole cell lysis. Taken together, these results suggest that RI18 has potential for further therapeutic research against frequently-encountered bacteria and fungi. Meanwhile, modification of AMPs is a promising strategy for developing novel antimicrobials to overcome drug-resistance.

The wide use of antibiotics in the past decades has driven the emergence of multidrug-resistant microbes at an unprecedented rate. In the Global Risks 2014 report, antibiotic-resistance was listed as one of thirty-one global risks related to social stability^1^.

Antimicrobial peptides (AMPs) found in host immune system have been proposed as new anti-infectives to replace or accompany conventional antibiotics due to their broad-spectrum antimicrobial activity and unique membrane-action mechanism. In contrast to the majority of available antibiotics that target specific biosynthetic pathways, it is metabolically costly for most microbes to develop resistance to AMPs by mutating or repairing membrane components^2^,^3^. As typical amphipathic peptides, α-helical peptides have been widely investigated in recent years for clinical trial development^4^,^5^,^6^. However, the application of α-helical AMPs has been hindered by some serious obstacles, such as systemic toxicities and high manufacturing costs. To address these issues, short AMPs derivatives with cell-selective toxicity have been developed based on the amino acid composition, charge, and hydrophobicity of natural AMPs^7^,^8^,^9^,^10^,^11^.

Porcine myeloid antimicrobial peptide-36 (PMAP-36) is a cathelicidin-related antimicrobial peptide deduced from porcine myeloid cDNA with an amphipathic α-helical conformation at the N-terminus^12^. Of all 11 porcine cathelicidins, PMAP-36 contains the highest net positive charge, which is considered an important parameter for peptide activity^13^,^14^. Previous studies have indicated that full-length PMAP-36 has potent antimicrobial activity^12^,^15^ and high toxicity^15^. To reduce its toxicity, we previously synthesized a series of PMAP-36 derivatives and characterized their antimicrobial and hemolytic activity. GI24, a 24-residue α-helical peptide truncated from the N-terminus of PMAP-36, retained high antibacterial activity, suggesting that the N-terminal domain of PMAP-36 is its active center. In addition, deletion of the random coil C-terminal eliminated the hemolysis caused by PMAP-36^15^. To develop shorter peptides in favour of reducing toxicity and synthesis cost, a series of derivatives were further designed in this study according to the amino acid sequence and structure of GI24. Effects of hydrophobicity and chain length on biological activities were evaluated, and the peptide with the greatest cell selectivity was identified.

Previous studies have demonstrated the *in vitro* antifungal activities of other α-helical cathelicidin peptides, including sheep myeloid antimicrobial peptide-29 (SMAP-29)^16^ and bovine myeloid antimicrobial peptide-28/27 (SMAP-28/27)^17^,^18^. Our previous study reported the potent activity of PMAP-36 and its analogs against gram-negative and gram-positive bacterial strains^15^, while the antifungal effects and potential mechanisms are poorly understood. Therefore, in addition to determining antibacterial activities, we measured the *in vitro* antifungal properties of PMAP-36 and its derivatives against *Candida* in the present study. *C. albicans* is a common opportunistic high-infective fungus and was used as a reference fungal strain to investigate the antifungal mechanism of AMPs against *Candida*^19^,^20^,^21^.

In the current study, melittin was used as a reference peptide to evaluate the biological activity and cell selectivity of the peptides tested. Melittin is a 26-residue antimicrobial peptide derived from bee venom and is commonly used as a cell-lysis and membrane-target peptide against pathogens and eukaryotic cells^22^,^23^,^24^. In this study, hemolytic activity of peptides was determined as readout of toxicity against mammalian cells. The selectivity index (SI) was calculated to assess the cell selectivity of peptides. Peptide serum stability was determined using killing kinetics assay. Flow cytometric, field emission-scanning electron microscopy (FE-SEM), and transmission electron microscopy (TEM) assays were employed to investigate the potential membrane destruction mechanisms of the peptides against bacteria and fungi. The overall aim of this study was to develop AMPs modification strategies to generate shorter peptide analogs with improved antimicrobial activities and cell selectivity.

## Results

### Peptide design and physicochemical parameters

**Figure 1:**
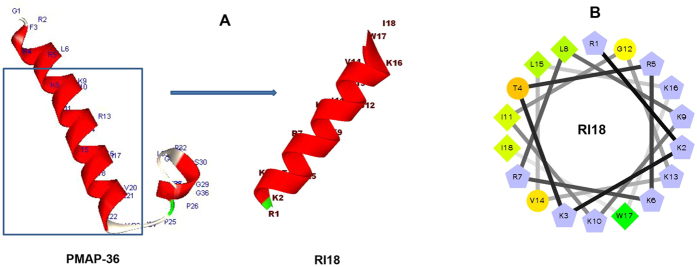
(**A**) Peptides three-dimensional structure projections of PMAP-36 and RI18. (**B**) Helical wheel projections of RI18. By default the output presents the hydrophilic residues as circles, hydrophobic residues as diamonds, and potentially positively charged as pentagons. Hydrophobicity is color coded as well: the most hydrophobic residue is green, and the amount of green is decreasing proportionally to the hydrophobicity, with zero hydrophobicity coded as yellow. Hydrophilic residues are coded red with pure red being the most hydrophilic (uncharged) residue, and the amount of red decreasing proportionally to the hydrophilicity. The potentially charged residues are light blue.

The peptides in the present study were designed based on the peptide PMAP-36. As shown in Fig. 1, PMAP-36 contains a typical α-helical conformation at the N-terminus. Our previous study indicated that GI24, the N-terminal 24-residue peptide form PMAP-36, has strong antimicrobial activity against gram-negative and gram-positive bacteria. The primary structure showed that most of hydrophobic residues were located at the C-terminal of GI24, and increasing hydrophobicity in a certain range has been known to improve antimicrobial activity^25^. Our previous study showed similar results. The high hydrophobicity amino acid (Trp) at position 23 of GI24 played an important role in guaranteeing its high antimicrobial activity^15^. Therefore, in this study, the high hydrophobic region was preserved in designing new peptide analogs. We evaluated the effects of chain length and hydrophobicity on biological activities by deleting three amino acids at a time at the N-terminus of GI24 and keeping the net charge equal or higher than +6, which is thought to be the threshold for driving peptides to the microbial membrane surface via electrostatic attraction^26^,^27^.

The theoretical calculated and measured molecular weights and key physicochemical parameters of the peptides are summarized in Table 1. The measured molecular weights of all the peptides were consistent with their theoretical values, suggesting that the peptides were successfully synthesized. After modification, the chain lengths of the derivatives ranged from 21 to 12 residues, with net charge from +13 to +6. Meanwhile, the hydrophobicity was ranked as follows: RI21 < RI18 < TI15 < RI12.

### Circular dichroism (CD) spectra

**Figure 2:**
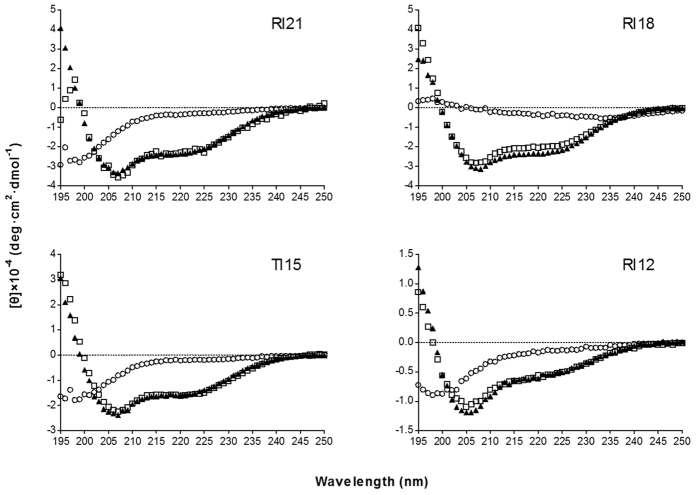
CD spectra of the peptides. The peptides were dissolved in 10 mM sodium phosphate buffer (pH 7.4) (⚪, circles), 50% TFE (▲, triangles), or 30 mM SDS (◻, squares). The mean residue ellipticity was plotted against wavelength. The values from three scans were averaged per sample. The peptide concentrations were fixed at 150 μM.

The secondary structure of four derivatives was investigated in different environments, 10 mM PBS (pH 7.4, mimicking aqueous environment), 50% trifluoroethanol (TFE, mimicking the hydrophobic environment of the microbial membrane), and 30 mM SDS (mimicking negatively charged prokaryotic membrane environment) by CD spectroscopy. As shown in Fig. 2, all the peptides displayed random coil conformations in 10 mM PBS. In the presence of 50% TFE and 30 mM SDS, the spectrums of peptides were characteristic of α-helix conformations, as indicated by the presence of two negative dichroic bands at approximately 208 and 222 nm. The amino acids distribution of RI18 in the secondary structure was predicted by the helical wheel projection (Fig. 1). RI18 displayed an amphipathic structure with opposing hydrophobic and polycationic surfaces.

### Antibacterial activity

The antimicrobial activities of the parental peptide and shorter derivatives against a panel of bacteria are summarized in Table 2. RI21 and RI18 retained the high antibacterial activity of PMAP-36, with the minimum inhibitory concentration (MIC) ranging from 1 to 8 μM across bacterial species. However, TI15 and RI12 exhibited a dramatic decrease in antimicrobial activity, especially against *S. aureus* 29213, *S. aureus* 25923, and *S. epidermidis* 12228, with more than 16-fold higher MIC values compared to PMAP-36. All peptides investigated displayed high antibacterial potency against *B. subtills*, with MIC at 1–2 μM. The minimum bactericidal concentration (MBC) values of the peptides were equal or 2–4 times higher than MIC values.

### Antifungal activity

The antifungal activities of the peptides were further determined against common infective *Candida* strains, MIC and the minimum fungicidal concentration (MFC) values are summarized in Table 3. The PMAP-36 parental peptide exhibited weak antifungal activity against *C. albicans*, while reducing the chain length improved the antifungal activity. RI21 and RI18 were the most effective derivatives against *C. albicans* 2.2086 and clinical isolated *C. albicans* CA276, with MIC of 8 and 16 μM, respectively. However, further reduction of chain length compromised the activity, as observed for TI15 and RI12. All the peptides were extremely active against *C. tropicalis* 2.1975, with MIC ranging from 1 to 8 μM, which was comparable to melittin. RI21 and RI18 also displayed higher antifungal efficiency against *C. krusei* 2.1857 than PMAP-36. The antibiotics, with the exception of fluconazole against *C. krusei*, exhibited higher potency against fungi than the peptides. The MFC values were equal or 2–4 times higher than MIC values across fungal species.

### Hemolytic activity

The hemolytic activity of the peptides against human erythrocytes was measured to assess systemic toxicity of the peptides against mammalian cells (Table 4). All shorter derivatives showed lower toxicity compared to PMAP-36. TI15 and RI12 did not show hemolytic activity even at the highest tested concentration of 128 μM. PMAP-36, RI21, and RI18 induced 5% hemolysis at 4, 32, and 128 μM, respectively. In contrast, melittin caused 5% hemolysis at a low concentration of 0.25 μM.

### Cell selectivity

The geometric mean (GM) of MIC values was calculated to reflect the therapeutic effect of the peptides across different pathogen species (Table 4). PMAP-36 showed the highest activity against bacteria than any of tested peptides. RI21 and RI18 were more active than melittin against bacteria, with GM of 2.00 and 2.38 μM, respectively. RI21 exhibited the lowest GM value against *Candida*. RI18 also showed high activity against *Candida*, with GM of 5.66 μM, which was approximately 3 times lower than that of PMAP-36. TI15 and RI12 displayed moderate activity against both bacteria and fungi.

SI was defined as the ratio of the minimal hemolytic concentration (MHC) to GM and was calculated to assess cell selectivity of the peptides. As show in Table 4, RI18 displayed the highest SI against both bacteria and fungi, at 53.78 and 22.61, respectively, which were approximately 19- and 108-fold improvement as compare to the parental peptide PMAP-36. Melittin showed the lowest cell selectivity against both bacteria and fungi with SI of 0.11 and 0.07, respectively.

### Effects of serum on antimicrobial activity

**Figure 3:**
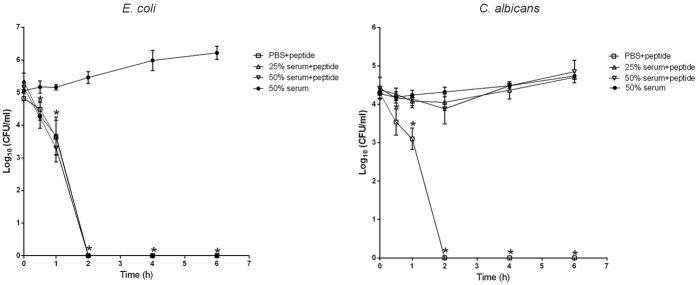
Effect of serum on killing kinetics of RI18 at 2 × MIC against *E. coli* (left) and *C. albicans* (right). Data were analyzed by ANOVA using SPSS 16.0 software. *P < 0.05, compared to the value of 50% serum at the same point in time. Data are means ± standard deviations of three independent experiments.

The stability of RI18 antimicrobial activity in the presence of serum was evaluated using a time-killing assay. As shown in Fig. 3, RI18 was bactericidal against *E. coli* within 2 h at a concentration of 2 × MIC in PBS. The killing kinetics of RI18 in 25% or 50% serum did not change from that in PBS with a 5-log_10_ bacterial reduction (99.9% clearance) within 2 h. However, the presence of serum diminished the killing efficacy of RI18 against *C. albicans*.

### Flow cytometry

**Figure 4:**
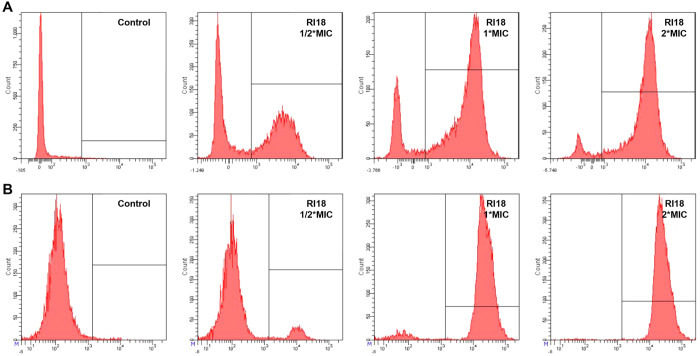
Flow cytometric analysis of *E. coli* (**A**) and *C. albicans* (**B**) treated with RI18. The increments of cellular fluorescence intensity of PI (10 μg/mL) after treating with the peptides was analyzed by flow cytometry.

The DNA intercalating dye propidium iodide (PI) was used as a comprehensive indicator to investigate cell membrane integrity and cell death by flow cytometry (Fig. 4). In the absence of peptide, the percentage of *E. coli* ATCC 25922 and *C. albicans* 2.2086 cells with PI fluorescent signal was only 0.8% and 1.4%, respectively, indicating viable cell membranes. After treatment with different concentrations of RI18, the percentage of PI-positive *E. coli* increased to 51.2% (1/2 × MIC), 80.3% (1 × MIC), and 91.6% (2 × MIC). For *C. albicans*, RI18 treatment resulted in 9.5% (1/2 × MIC), 93.5% (1 × MIC), and 99.0% (2 × MIC) positive nucleic acid staining. A dose-dependent increase in PI fluorescence indicated that RI18 was capable of damaging the *E. coli* and *C. albicans* cell membrane.

### FE-SEM

**Figure 5:**
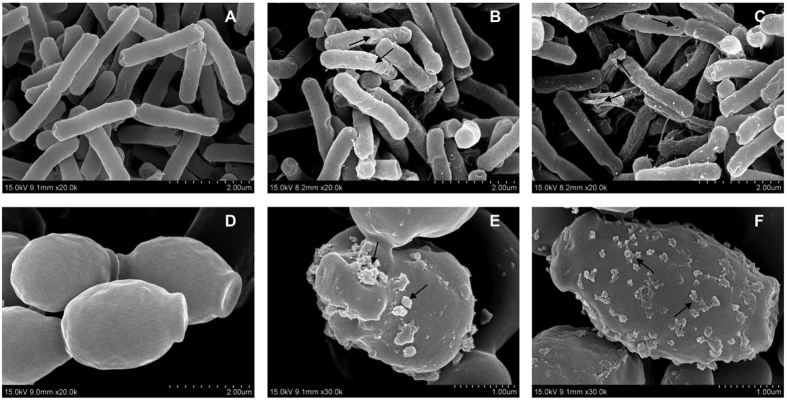
Scanning electron microscopic micrographs of *E. coli* and *C. albicans* treated with RI18. FE-SEM micrographs of *E. coli*: (**A**) control, without peptide; (**B**) RI18 at 1 × MIC; (**C**) RI18 at 2 × MIC. FE-SEM micrographs of *C. albicans*: (**D**) control, without peptide; (**E**) RI18 at 1 × MIC; (**F**) RI18 at 2 × MIC. Exponential phase *E. coli* and *C. albicans* cells were treated with different concentrations of RI18 for 1 h.

A direct visualization of peptide-induced *E. coli* and *C. albicans* cellular damage was evaluated by FE-SEM. Fig. 5B,C shows the FE-SEM images of *E. coli* after treatment with RI18 at 1 × MIC or 2 × MIC for 1 h. Control cells not treated with peptide exhibited bright and smooth surfaces (Fig. 5A), but RI18 treatment induced cell morphological changes. The membrane surfaces of *E. coli* cells became roughening and corrugating upon 1 × MIC RI18 treatment, the membrane became more rough and ruptured following 2 × MIC RI18 treatment.

We observed similar effects of RI18 on C. *albicans* membrane (Fig. 5D–F). Compared to the control, 1 × MIC RI18 treatment induced partial membrane damages and the formation of discrete blebs (Fig. 5E). Moreover, treatment with 2 × MIC peptide induced increased membrane surfaces roughness and blebbing (Fig. 5F).

### TEM

**Figure 6:**
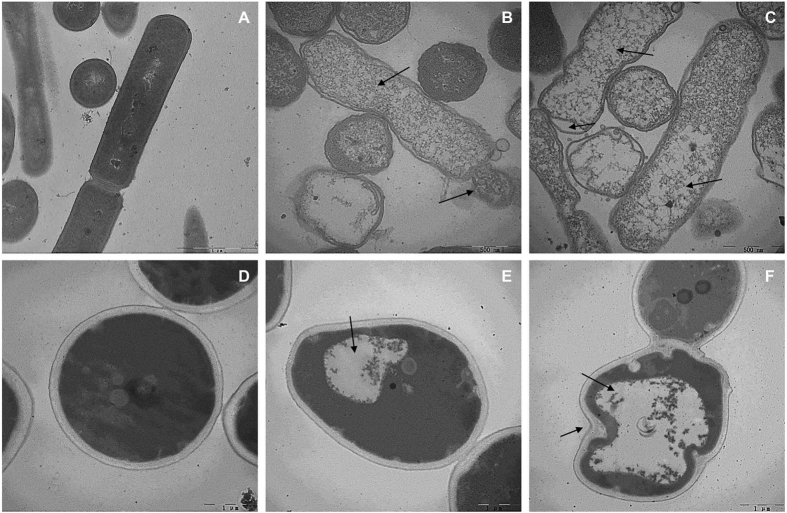
Transmission electron microscopic micrographs of *E. coli* and *C. albicans* treated with RI18. TEM micrographs of *E. coli*: (**A**) control, without peptide; (**B**) RI18 at 1 × MIC; (**C**) RI18 at 2 × MIC. TEM micrographs of *C. albicans*: (**D**) control, without peptide; (**E**) RI18 at 1 × MIC; (**F**) RI18 at 2 × MIC. Exponential phase *E. coli* and *C. albicans* cells were treated with different concentrations of RI18 for 1 h

TEM analysis was employed to observe the ultrastructural alterations in *E. coli* and *C. albicans* after 1 × MIC or 2 × MIC RI18 treatment. Control *E. coli* culture showed complete cell membrane and homogeneous cytoplasm (Fig. 6A). In contrast, 1 × MIC RI18 induced significant rupture of cell membrane, release of cellular contents, and obvious cytoplasmic clear zones (Fig. 6B). In addition, the cytoplasmic membrane of most *E. coli* cells were irregular and separated from the outer cell membrane after 2 × MIC RI18 treatment for 1 h, and the dispersion of the intracellular contents was observed (Fig. 6C).

Fig. 6D–F shows the effects of RI18 on *C. albicans*. Untreated *C. albicans* cells exhibited dense cytoplasm enveloped by a typical cell wall structure (Fig. 6D). Compared to the control, 1 × MIC RI18 treatment decreased cytoplasmic volume and induced the formation of a large cytoplasmic vacuole, but had no apparent effect on the cell wall (Fig. 6E). After 1 h treatment with 2 × MIC RI18, vacuole expansion and further reduction of intracellular contents were observed, whilst cell wall became irregular (Fig. 6F).

## Discussion

AMPs have attracted attention as promising therapeutic alternatives to conventional antibiotics due to their broad-spectrum antimicrobial activities and unique mode of action against pathogens. However, high synthesis cost and systemic toxicity have been challenges for the further application of natural peptides. Recently, modification of natural AMPs has been shown to be an effective strategy for reducing the manufacturing cost and limiting the toxicity^15^,^28^,^29^,^30^. In our previous study, we reported an α-helical peptide derived from the natural peptide PMAP-36 by deleting the unstructured C-terminus. Biological activity assays showed that a short peptide analog (GI24) retained the high antimicrobial potency and limited the hemolytic activity caused by PMAP-36. This typical α-helical analog provided a good template for researching quantitative structure-activity relationships (QSARs) of α-helical peptides. In addition, we characterized the crucial site of GI24 using single residue substitution. A high hydrophobic residue at the C-terminus of GI24 plays an important role in killing pathogens^15^. Therefore, in the current study, the high hydrophobic C-terminus of GI24 was preserved and a series of shorter peptides were generated by deleting three N-terminal amino acids at a time to determine the effects of the chain length and hydrophobicity on the biological activities of the peptides.

As expected, all the peptides folded into amphipathic α-helix conformations in the membrane-mimetic environments (50% TFE and 30 mM SDS). Previous studies have shown that the conformational transformation from unstructured conformations in the aqueous environment to the amphipathic α-helix in the membrane environment played a crucial role in the peptide partition to the membrane and correlated with antimicrobial activity^6^,^31^,^32^,^33^. After the initial electrostatic attraction and aggregation, the peptide moved from the aqueous environment to the membrane environment, followed by a conformational transformation and correct orientation. The peptides then rearrange and partition into the membrane^14^,^32^,^34^.

The crucial physicochemical parameters of peptides were calculated by using online tools. As shown in Table 1, the hydrophobicity of the derivatives ranged from −3.10 (RI21) to −0.11 (RI12), and the net charge varied from +13 to +6. In this study, increasing hydrophobicity did not improve antimicrobial activity. For net charge, previous studies have shown that the cationic portions of AMPs facilitate the initial electrostatic attraction and drive the peptide to negatively charged components on the microbial membrane surface^33^,^35^,^36^. However, later studies demonstrated that the relationship between charge and antimicrobial activity was non-linear and that above a certain threshold (usually +6), the increasing positive charge did not improve antimicrobial activity^26^,^27^. In this study, the net charges of the derivatives were all above the +6 threshold, suggesting adequate initial electrostatic attraction to drive the peptides to the membrane and a weaker relationship between the change in charge and antimicrobial activity. In addition to hydrophobicity and charge, chain length is thought to be another critical factor that correlates with AMPs antimicrobial activity. The amino acid length required for the peptides to span the membrane is approximately 15–20 residues, which is slightly change depending on the thickness of the bilayer^32^,^37^,^38^. In this study, antimicrobial activity assays demonstrated that RI21 and RI18 retained the high antibacterial activities of the parental peptide PMAP-36 against bacteria. However, the activities of the peptides decreased as chain length decreased, especially when the number of residue was lower than 15. These data suggest that the particular peptide chain length was required for spanning the membrane.

It is noteworthy that RI21 and RI18 also showed excellent antifungal activity. In recent years, the challenge of clinical fungal infections has raised concerns due to the lack of effective antifungal agent. AMPs have been acknowledged as a novel class of antifungal agents that may be used to treat fungal infections. Therefore, in addition to examining the antibacterial activity, the antifungal properties of PMAP-36 and its derivatives were further determined against highly pathogenic fungi. *C. krusei* and *C. tropicalis* were susceptible to all the peptides investigated in this study, with MIC ranging from 1 to 16 μM. The activity of the derivatives initially increased and then decreased against *C. albicans*, as chain length decreased. Moderate length peptides (approximately 20 residues), such as RI21 and RI18 displayed high activity against *C. albicans*. According to the GM values, the derivatives were more effective against bacteria than *Candida* species. This could be due to the differences in cell membrane composition.

To verify the safety of these compounds, the toxicity of the peptides against mammalian cells was evaluated by determining their erythrocyte lysis ability. Hemolytic activity decreased as chain length decreased, which is consistent with previous findings^39^,^40^. Taking antimicrobial activity into consideration, RI18 exhibited the greatest selective toxicity towards pathogens over host cells. In order to evaluate the cell selectivity, this index has been quantified by calculating the SI, and larger SI values indicate greater cell selectivity^41^. RI18 displayed the highest SI value against bacteria (53.78), which was approximately 19 and 489 times higher than that of PMAP-36 and melittin, respectively. These results emphasized the effectiveness of peptide modification. It is noteworthy that although both fungi and host cells are eukaryotic cells, RI18 still showed high selective toxicity towards fungi over host cells with an SI of 22.61, indicating its wider therapeutic window for systemic applications. The selectivity could be attributed to the different membrane lipid components in fungi and mammalian cells. Ergosterol is the main lipid component of fungi, while cholesterol is the main lipid in mammalian cells^42^.

Based on the hemolytic and antimicrobial activity results, the RI18 peptide was the most promising derivative among all the peptides presented in this study. To assess the stability of RI18 in body fluids, its antimicrobial activity was tested in the presence of human serum, a complex body fluid known to inhibit AMPs activity^11^,^43^. In the presence of 25% or 50% serum, RI18 maintained its antibacterial efficiency against *E. coli* but lost activity against *C. albicans*. AMPs may be inactivated by body fluids (e.g. serum) because of blood components binding, proteases degradation or competition for membrane binding sites^43^. We have previously noted different inhibitory effect across microbial species^44^,^45^. It can be argued that the differential susceptibilities to RI18 of bacteria and fungi may be due to some serum components preferentially bind to the fungal surfaces, hampering the interaction of peptide with binding sites.

We then further investigated the mode of action of RI18 in killing bacterial and fungal cells. The antibacterial mechanism of PMAP-36 and its analogs was to damage the cell membrane, ultimately resulting in cell death^15^,^46^. In addition, previous studies reported that most α-helical antifungal peptides exerted antifungal activity by disrupting membrane integrity^47^,^48^. Hence, in the current study, the antimicrobial mechanism study was investigated with a particular focus on the effects of RI18 on cell membranes. For bacteria, the first step of peptide-membrane interaction was considered peptide binding to the negatively charged components of the outer membrane such as lipopolysaccharide (LPS), followed by the membrane destabilization^13^,^49^. Fungi display different cell organization compared to bacteria, possessing both cell membrane and cell wall. In addition, the cell wall primarily consists of glucan, mannan and chitin^50^. Previous study has demonstrated that AMPs can bind to glucan, and its binding activity affected its antifungal activity. After binding, the peptides can pass though the cell wall and interrupt the cell membrane^42^. Therefore, the membrane permeability was first investigated by monitoring PI fluorescence, which is a DNA-staining fluorescent dye that indicates compromised cell membrane and cell death^51^,^52^. The rapid increase in the percentage of PI-positive cells revealed that RI18 both damaged the membranes of *E. coli* and the *C. albicans* in a dose-dependent manner. RI18 possessed the ability to destroy the cell membrane, which was further confirmed by FE-SEM and TEM analyses. As evidence of large lesions, shriveling or blebbing on the cell surface and leakage of cellular contents were observed in both *E. coli* and *C. albicans*, suggesting that RI18 killed the microorganisms primarily via membrane disruption.

## Conclusion

In this study, we designed a series of short peptides by modifying PMAP-36. All of the peptides displayed the typical α-helical structure in the membrane-mimetic environment. These peptides exhibited excellent antimicrobial activity against both gram-positive and gram-negative bacteria. The moderate-length peptides (approximately 20 residues) displayed the highest activity against *C. albicans*. Meanwhile, the modification limited systemic toxicity and improved cell selectivity. The RI18 derivative showed the greatest cell selectivity among these peptides, and its antibacterial efficacy against *E. coli* was not affected by serum. The results from flow cytometry, FE-SEM and TEM assays revealed that RI18 killed bacteria and fungi primary by damaging the cell membrane integrity, leading to cytosol leakage and ultimate pathogen death. The findings reported herein suggest that modification of natural active peptides is a simple and feasible approach for developing novel antimicrobial agents with enhanced cell selectivity, and the RI18 peptide holds potential as a promising antimicrobial agent for further biotechnological and clinical applications.

## Methods

### Peptide design and sequence analysis

Primary peptide sequence analysis was performed using bioinformatics programs ProtParam (ExPASy Proteomics Server: http://www.expasy.org/tools/protparam.html). The mean peptide hydrophobicity was calculated online using CCS scale (http://www.bbcm.univ.trieste.it/~tossi/HydroCalc/HydroMCalc.html). The helical wheel projection was calculated online using Helical Wheel Projections (http://rzlab.ucr.edu/scripts/wheel/wheel.cgi). The three-dimensional structure was predicted online by I-TASSER (http://zhanglab.ccmb.med.umich.edu/I-TASSER/).

### Peptide synthesis

All peptides listed in Table 1 were synthesized by GL Biochem (Shanghai, China) with solid-phase methods using N-(9-fluorenyl) methoxycarbonyl (Fmoc) chemistry. Previous studies have indicated that C-terminal amidation strongly correlated to antimicrobial activity^53^,^54^ and stability^55^. Therefore, all peptides investigated in this study were amidated at the C-terminus. Matrix-assisted laser desorption/ionization time-of-flight mass spectroscopy (MALDI-TOF MS, Linear Scientific Inc., USA) was used to identify the mass of these peptides. The purity of peptides was confirmed as higher than 95% using analytical reverse-phase high-performance liquid chromatography (RP-HPLC). Peptides were then dissolved in DI water at a concentration of 2.56 mM and stored at −20 °C before subsequent assessments.

### CD analysis

The secondary structures of the peptides in different environments were measured using a J-820 spectropolarimeter (Jasco, Tokyo, Japan). The spectra were recorded at a scanning speed of 10 nm/min at wavelengths ranging from 195 to 250 nm in sodium phosphate buffer (10 mM, pH 7.4), SDS micelles (30 mM, Sigma), or TFE (50%, Sigma). An average of three scans was collected for each peptide. The final concentration of the peptides was 150 μM.

The acquired CD signal spectra were converted to the mean residue ellipticity using the following equation:


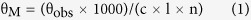


where θ_M_ is the mean residue ellipticity [deg.cm^2^.dmol^−1^], θ_obs_ is the observed ellipticity corrected for the buffer at a given wavelength [mdeg], c is the peptide concentration [mM], l is the path length [mm], and n is the number of amino acids.

### Antibacterial activity assays

The peptide antibacterial activity was determined against both gram-positive and gram-negative bacteria strains, including *Escherichia coli* ATCC 25922, *Escherichia coli* UB1005, *Salmonella enterica* serovar typhimurium C77-31, *Staphylococcus aureus* ATCC 29213, *Staphylococcus aureus* ATCC 25923, *Bacillus subtilis* CMCC 63501, and *Staphylococcus epidermidis* ATCC 12228. Clinically isolated *Escherichia coli* EC183 was provided by the 4th Affiliated Hospital of Harbin Medical University. MIC of the peptides was measured using a modified version of the National Committee for Clinical Laboratory Standards (NCCLS) broth microdilution method as described previously^56^. The bacterial strains were inoculated and grown to mid-log phase in fresh Mueller-Hinton broth (MHB) at 37 °C. Bacterial inoculum suspensions were prepared at a final concentration of approximately 1 × 10^5^ CFU/ml. Peptides were 2-fold serially diluted to make different concentrations, from 0.5 to 256 μM. Equal volumes of inoculum suspensions were then added to each well of a sterile 96-well plate with different concentrations of peptides, and the plate was incubated for 18 h at 37 °C. Broth with or without bacteria was employed as the positive control or the negative control, respectively. MIC was defined as the lowest concentration of peptides that prevented visible turbidity by visual inspection. MBC was further determined followed the MIC incubation period by transferring 10 μl samples from each well that did not show visible bacterial growth and plating on Mueller-Hinton agar (MHA) plates. After overnight incubation at 37 °C, MBC was defined as the lowest concentration of peptides that killed at least 99.9% of the initial inoculums. Experiments were performed in triplicate with three biological replicates.

### Antifungal susceptibility testing

The antifungal activities were monitored in RPMI-1640 broth medium by a 2-fold microdilution assay according to the Clinical and Laboratory Standards Institute (CLSI) methods^57^. The *Candida albicans* CGMCC 2.2086, *Candida tropicalis* CGMCC 2.1975, and *Candida krusei* CGMCC 2.1857 fungal strains were purchased from the China General Microbiological Culture Collection Center (CGMCC, Beijing, China). Clinically isolated *Candida albicans* CA2761 was provided by the 4th Affiliated Hospital of Harbin Medical University. Briefly, fungal strains were cultured on YPD agar plates (1% peptone, 0.5% yeast extract, 1% glucose and 2% agar) at 28 °C to prepare *Candida* suspensions with a turbidity of a 0.5 McFarland standard. A total of 50 μl inoculum cell suspension diluted in RPMI-1640 broth medium was added to an equal volume of peptide solution to achieve a final peptide concentrations ranging from 0.25 μM to 128 μM in a 96-well plate. Pure broth alone or with inoculum suspensions was used as the negative control or the positive control, respectively. The plate was incubated at 28 °C for 48 h. MIC was considered the lowest concentration at which no fungal growth was visually observed under experimental conditions. MFC was measured by removing 10 μl samples from each well that did not show any visible fungal growth and plating on YPD agar plates. After 48 h incubation at 28 °C, MFC was defined as the lowest concentration of peptides that killed at least 99.9% of the initial inoculums. Experiments were performed in triplicate with three biological replicates.

### Quantification of hemolytic activity

The hemolytic activity of the peptides was measured as the amount of hemoglobin released by the lysis of human erythrocytes^58^. The experimental protocol was reviewed and approved by the ethics committee of the Northeast Agricultural University Hospital, and the experimental method was carried out in accordance with the approved guidelines and regulations. Briefly, fresh and healthy human blood cells (hRBCs) in a polycarbonate tube containing heparin were centrifuged at 1,000 × g for 5 min at 4 °C. The erythrocytes obtained were washed three times and re-suspended in PBS. Then, the erythrocyte solution of 50 μl was incubated with 50 μl of the respective peptides dissolved in PBS for 1 h at 37 °C. Intact erythrocytes were pelleted by centrifugation at 1,000 × g for 5 min at 4 °C, and the supernatant was transferred to a new 96-well plate. Hemoglobin release was monitored using an absorbance microplate reader (TECAN GENios F129004; TECAN, Austria) at 570 nm. hRBCs in PBS and 0.1% Triton X-100 were employed as negative and positive controls, respectively. MHC was defined as the minimal peptide concentration that caused 5% hemolysis. Three independent experiments were performed in duplicate.

### Selectivity index calculation

SI values were determined by the ratio of MHC to GM, indicating the specificity of peptides towards pathogens and eukaryotic cells. When no antimicrobial activity was observed at 128 μM, a value of 256 μM was used to calculate the GM values. For the hemolytic activity, 256 μM was used when no hemolysis was observed at 128 μM, the highest tested concentration. Larger SI values indicate greater cell selectivity.

### Effects of serum on antimicrobial activity

Blood was drawn from the antecubital veins of three healthy donors into glass tubes without anticoagulant and spontaneously allowed to clot at room temperature for 1 h. The experimental protocol was reviewed and approved by the ethics committee of the Northeast Agricultural University Hospital, and the experimental method was carried out in accordance with the approved guidelines and regulations. Serum was collected after centrifugation for 10 min at 1,000 × g followed by heat inactivation at 56 °C for 30 min. Exponentially growing *E. coli* 25922 was washed three times at 1,000 × g for 10 min with PBS and diluted in PBS or 25% and 50% serum to a final density of 10^6^ CFU/ml. The cell suspensions were incubated with 2 × MIC RI18 at 37 °C for 6 h. Aliquots (50 μl) were removed from the samples at specific intervals (0, 0.5, 1, 2, 4, and 6 h), serially diluted in PBS buffer and plated on MHA plates. Colonies were counted after 24 h incubation at 37 °C.

*C. albicans* 2.2086 suspensions diluted in buffer with or without serum were incubated with 2 × MIC RI18 at 28 °C. Samples were removed at specific intervals (0, 0.5, 1, 2, 4, and 6 h), serially diluted and plated on YPD plates. Following incubation for 48 h at 28 °C, colonies were counted. Each experiment was performed three independent times.

### FACScan analysis

The integrity of the cell membranes after peptide treatment was determined by flow cytometry according to a previously described method^59^. *E. coli* 25922 and *C. albicans* 2.2086 were cultured to mid-log phase and harvested by centrifugation at 1,000 × g for 10 min. The cells were washed thrice with 10 mM PBS and diluted to 10^5^ CFU/ml in the same buffer. The desired concentration of the peptide was added, and the mixture was incubated for 30 min at 28 °C with constant shaking at 140 rpm. PI (final concentration of 10 μg/ml, Sigma) was added and incubated for an additional 30 min at 4 °C. At the end of the incubation, the unbound dye was removed by washing with an excess of PBS. *E. coli* cells incubated with PI in the absence of peptide treated served as a negative control. The data were recorded using a FACScan instrument (Bectone-Dickinson, San Jose, CA) at a laser excitation wavelength of 488 nm.

### FE-SEM analysis

*E. coli* 25922 and *C. albicans* 2.2086 were cultured to mid-log phase. The cells were harvested by centrifugation at 1,000 × g for 10 min, washed thrice with 10 mM PBS and re-suspended to an OD_600_ of 0.2. The cell suspension was incubated at 37 °C for 60 min with different peptides at their 1 × MIC or 2 × MIC. Following the incubation, the cells were centrifuged and washed with PBS 3 times at 5,000 × g for 5 min. Microbial cell pellets were then fixed overnight with 2.5% (v/v) glutaraldehyde in PBS at 4 °C and washed twice with PBS. Thereafter, the cell pellets were dehydrated in a graded ethanol series (50%, 70%, 90%, and 100%), for 10 min each. The dried samples were transferred to a mixture (1:1, v/v) of ethanol and tertiary butanol for 20 min, followed by pure tertiary butanol for 1 h. The specimens were dried using a critical point dryer, coated with gold, and visualized under a field emission scanning electron microscope (Hitachi S-4800, Japan).

### TEM analysis

The microbial sample was initially prepared as described above for FE-SEM analysis. After pre-fixation with 2.5% glutaraldehyde overnight, the cell pellets were washed 3 times with PBS and post-fixed with 2% osmium tetroxide in PBS for 70 min. The samples were washed twice with PBS, followed by dehydration for 9 min in a graded ethanol series (50%, 70%, 90%, and 100%), and incubated for 10 min each in 100% ethanol, a mixture (1:1) of 100% ethanol and acetone, and absolute acetone. These samples were then transferred to a 1:1 mixture of absolute acetone and epoxy resin for 30 min, and then immersed in pure epoxy resin in a constant-temperature incubator overnight. Finally, specimens were sectioned using an ultramicrotome, stained with uranyl acetate and lead citrate, and observed using a transmission electron microscope (HITACHI H-7650, Japan).

### Statistical analysis

Data were analyzed by ANOVA using SPSS 16.0 software. The data are presented as the means ± standard deviation. The statistical significance was defined as a *P*-value of less than 0.05.

## Additional Information

**How to cite this article**: Lyu, Y. *et al*. Antimicrobial activity, improved cell selectivity and mode of action of short PMAP-36-derived peptides against bacteria and *Candida. Sci. Rep.* **6**, 27258; doi: 10.1038/srep27258 (2016).

## Figures and Tables

**Table 1:** Amino acid sequences, formula, molecular weights, charge, and hydrophobicity values of the peptides used in this study.

**Peptides**	**Sequence**	**Formula**	**Theoretical MW**	**Measured MW** a	**Net Charge**	**H**^b^
PMAP-36^c^	GRFRRLRKKTRKRLKKIGKVLKWIPPIVGSIPLGCG-NH_2_	C_191_H_337_N_63_O_38_S_1_	4156.20	4156.27	+14	−1.41
RI21	RRLRKKTRKRLKKIGKVLKWI-NH_2_	C_124_H_231_N_45_O_22_	2704.40	2704.51	+13	−3.10
RI18	RKKTRKRLKKIGKVLKWI-NH_2_	C_106_H_196_N_36_O_19_	2278.90	2278.97	+11	−3.05
TI15	TRKRLKKIGKVLKWI-NH_2_	C_88_H_160_N_28_O_16_	1866.40	1866.43	+8	−1.67
RI12^c^	RLKKIGKVLKWI-NH_2_	C_72_H_129_N_21_O_12_	1480.90	1480.94	+6	−0.11

^a^Molecular weight (MW) was measured by MALDI-TOF mass spectroscopy.

^b^The mean hydrophobicity (H) was the total hydrophobicity of per residue of peptides calculated using CCS scale.

^c^PMAP-36 and RI12 physicochemical parameters were determined as previously described^15^.

**Table 2:** MIC and MBC of the peptides against bacteria.

**Peptides**	**MIC**^a^ **(MBC**^b^**) (μM)**							
	**Gram-negative bacteria**				**Gram-positive bacteria**			
	***E. coli 25922***	***E. coli EC183***	***E. coli 1005***	***S. typhi C77-31***	***S. aureus 29213***	***S. aureus 25923***	***S. epidermidis 12228***	***B. subtilis 63501***
PMAP-36	1^c^ (4)	1 (1)	2^c^ (2)	1^c^ (2)	2^c^ (4)	2^c^ (4)	2^c^ (4)	1 (1)
RI21	2 (8)	1 (2)	2 (2)	1 (2)	4 (4)	4 (4)	4 (8)	1 (4)
RI18	2 (4)	1 (2)	2 (4)	2 (4)	4 (4)	8 (8)	4 (16)	1 (2)
TI15	8 (16)	2 (4)	4 (8)	8 (32)	32 (128)	32 (128)	32 (64)	2 (4)
RI12	8^c^ (32)	2 (4)	16^c^ (32)	8^c^ (32)	64^c^ (128)	128^c^ (128)	64^c^ (64)	2 (8)
melittin	2^c^ (4)	2 (2)	2^c^ (4)	2^c^ (2)	8^c^ (16)	8^c^ (8)	0.5^c^ (1)	1 (2)

^a^Minimum inhibitory concentration (MIC) was determined as the lowest concentration of peptide that inhibited bacteria growth. Data are representative of three independent experiments.

^b^Minimum bactericidal concentration (MBC) was determined as the lowest concentration of peptide that killed at least 99.9% of the initial inoculums. Data are representative of three independent experiments.

^c^MIC values were determined as previously described^15^.

**Table 3:** MIC and MFC of the peptides against fungi.

**Peptides**	**MIC**^a^ **(MFC**^b^**) (μM)**			
	***C. albicans*** **2.2086**	***C. albicans CA276***	***C. tropicalis*** **2.1975**	***C. krusei*** **2.1857**
PMAP-36	128 (128)	128 (128)	1 (2)	8 (8)
RI21	8 (16)	8 (32)	1 (2)	2 (4)
RI18	16 (32)	8 (16)	2 (4)	4 (8)
TI15	64 (64)	128 (128)	2 (4)	8 (16)
RI12	128 (128)	128 (128)	8 (8)	16 (16)
melittin	4 (8)	8 (16)	2 (2)	2 (4)
Amphotericin B	0.25 (0.25)	0.5 (1)	0.25 (1)	1 (1)
Fluconazole	2 (2)	2 (2)	0.25 (1)	64 (64)

^a^Minimum inhibitory concentration (MIC) was determined as the lowest concentration of peptide that inhibited fungal growth. Data are representative of three independent experiments.

^b^Minimum fungicidal concentration (MFC) was determined as the lowest concentration of peptide that killed at least 99.9% of the initial inoculums. Data are representative of three independent experiments.

**Table 4:** MHC, GM and SI of the peptides.

**Peptides**	**MHC**^a^ **(μM)**	**GM**^b^ **(μM)**		**SI**^c^	
		**Bacteria**	**Fungi**	**Bacteria**	**Fungi**
PMAP-36	4	1.41	19.03	2.84	0.21
RI21	32	2.00	3.36	16	9.52
RI18	128	2.38	5.66	53.78	22.61
TI15	>128	8.72	19.03	29.36	13.45
RI12	>128	14.67	38.05	17.45	6.73
melittin	0.25	2.18	3.36	0.11	0.07

^a^MHC is the minimum hemolytic concentration that caused 5% hemolysis of human red blood cells (hRBCs). Data are representative of three independent experiments.

^b^The geometric mean (GM) of the MIC values of the peptides against bacteria and fungi was calculated.

^c^Selectivity index (SI) is the ratio of MHC to GM. Larger SI values indicate greater cell selectivity. When no detectable hemolytic activity was observed at 128 μM, a value of 256 μM was used to calculate SI.
